# Data Trustworthiness Evaluation in Mobile Crowdsensing Systems with Users’ Trust Dispositions’ Consideration

**DOI:** 10.3390/s19061326

**Published:** 2019-03-16

**Authors:** Eva Zupančič, Borut Žalik

**Affiliations:** Faculty of Electrical Engineering and Computer Science, University of Maribor, 2000 Maribor, Slovenia; borut.zalik@um.si

**Keywords:** data trustworthiness, human involvement, mobile crowdsensing, opinions, opportunistic sensing, participatory sensing, reputation systems, subjectivity, trust attitude, trust framework

## Abstract

Mobile crowdsensing is a powerful paradigm that exploits the advanced sensing capabilities and ubiquity of smartphones in order to collect and analyze data on a scale that is impossible with fixed sensor networks. Mobile crowdsensing systems incorporate people and rely on their participation and willingness to contribute up-to-date and accurate information, meaning that such systems are prone to malicious and erroneous data. Therefore, trust and reputation are key factors that need to be addressed in order to ensure sustainability of mobile crowdsensing systems. The objective of this work is to define the conceptual trust framework that considers human involvement in mobile crowdsensing systems and takes into account that users contribute their opinions and other subjective data besides the raw sensing data generated by their smart devices. We propose a novel method to evaluate the trustworthiness of data contributed by users that also considers the subjectivity in the contributed data. The method is based on a comparison of users’ trust attitudes and applies nonparametric statistic methods. We have evaluated the performance of our method with extensive simulations and compared it to the method proposed by Huang that adopts Gompertz function for rating the contributions. The simulation results showed that our method outperforms Huang’s method by 28.6% on average and the method without data trustworthiness calculation by 33.6% on average in different simulation settings.

## 1. Introduction

Smartphones and other smart devices have become an influential part of our everyday lives and one of the most powerful pervasive technologies. According to the Ericsson Mobility Report 2018 [1], there were 7.9 billion mobile subscriptions in Q1 2018, which is more than one for every person in the world. Around 60 percent of all mobile phone subscriptions are associated with smartphones, wherein  each active smartphone has, on average, 3.4 GB monthly data traffic.

Smartphones are not only ubiquitous, they also have powerful processors, integrated high-resolution cameras, a large number of different sensors, such as accelerometer, gyroscope, GPS, light sensors, proximity sensors, etc. Advanced sensing capabilities and the ubiquity of smart devices are the basis for the mobile crowdsensing paradigm. Mobile crowdsensing systems employ ordinary users (or citizens) to collect, monitor, process, store and share large amounts of data [2,3]. It extends participatory sensing approach with implicit data contribution and data extraction from other widely used applications [4]. User-generated content include opinions or experiences, which add knowledge to observed phenomenon importantly, but require additional consideration in data analysis. Collected data are certainly valuable to their owners for their own specific purposes (e.g., health monitoring), while providing benefits to the entire community. The most commonly proposed mobile crowdsensing systems scenarios are related to smart city services implementation (e.g., environmental pollution monitoring, smart traffic applications, trip planning, crime monitoring, noise monitoring, etc.) [5,6,7,8,9,10,11,12]. It requires different level of user involvement: implicit data capture, where data is collected through general, widely used applications (e.g., social media) and then extracted, or explicit data capture, where data are collected through purpose-made crowdsensing applications [13]. Furthermore, explicit data collecting can employ a participatory sensing approach, where individuals contribute sensor data actively, or the opportunistic sensing approach, where individuals participate in the capture of sensor data in the crowd-sensing system passively [14,15].

Since the success of mobile crowdsensing systems is based on a large number of voluntary participants who contribute data, such systems are exposed to erroneous or malicious data. Participants can post bad data inadvertently (for example, carrying a smartphone in a pocket when sampling data about street noise), or even deliberately (for example, posting fake reviews in order to get rewards). Both behaviors result in untrustworthy data, which need to be handled to ensure the reliability of applications and predictions that rely on the mobile crowdsensing approach. Trust and reputation management systems are common tools for assessing the trustworthiness of other participants and their contributions prior to using them for further analysis, used widely in online communities and mobile crowdsensing systems [16,17]. Despite the availability of different trust and reputation management systems proposals, they do often not consider human involvement properly and there remains a missing gap in this research area.

The objective of this work is to define a conceptual trust framework that considers human involvement in mobile crowdsensing systems and takes into account that users contribute their opinions and other subjective data besides the raw sensing data generated by their smart devices. Such users’ trust assessments are subjective and directly related to and affected by an individual’s perspective and situational factors [18,19,20]. The users are not aware neither what the other users’ factors, criteria or motivations are for their assessments and opinions, nor their own. Additionally, the  users can tamper with their observations before submitting them to a mobile crowdsensing system platform. Therefore, when collecting, analyzing and reporting data, mobile crowdsensing systems should take into account human factors and propose a suitable method that supplements methods for device generated data analysis. We take advantage of the fact that today’s smartphones are almost always and everywhere connected to the Internet and users can contribute data in an opportunistic way. We then combine the opportunistic and participatory sensing approaches and propose a novel trust framework and a method to evaluate the trustworthiness of data contributed by users, which focuses on an appropriate consideration of subjectivity in the contributed trust assessments and behavioral specifics of people.

The proposed conceptual framework and method are based on the findings derived from behavioral psychology and consider trust properties, such as irrationality and trust differentiation [21,22], and present a new approach to model trust in mobile crowdsensing systems.

The contributions of this paper can be summarized as follows:

- A novel conceptual trust framework for mobile crowdsensing systems that considers factors stemming from behavioral psychology and allows the proper treatment of subjective user contributions.
- An application-agnostic method for computing the trustworthiness of users’ contributions with data subjectivity consideration that uses nonparametric statistics instead of classifying users and does not require any explicit involvement of participants.
- An evaluation of the method’s efficiency under various settings in scenarios with different number of malicious users and in scenarios with different distributions of users’ personality types to show that the proposed method achieves lower error rate than the most relevant method for evaluating the trustworthiness of participants’ contributions in mobile crowdsensing systems, which also models dynamic human trust perception, proposed by Huang et al. [23].

The rest of the paper is organized as follows. The following section discusses related work. Section 3 describes our proposed trust framework for mobile crowdsensing systems, including a method for filtering untrustworthy user contributions. Section 4 describes the simulation environment and the design of our experiments. It also presents the obtained results and discusses further properties of the proposed trust framework. We provide concluding remarks in the last section.

## 2. Related Work

Trust and reputation management systems have been studied and used in different domains, such  as online markets [16,24], peer-to-peer networks [25], wireless sensor networks [18,26] and Internet of Things [27,28]. However, the mobile crowdsensing paradigm has specific characteristics, such as the involvement of humans in the sensing loop, which dictates the need for more adapted approaches. In recent years, several trust and reputation systems in the domain of mobile crowdsensing systems there have been proposed. However, the concept of trust in mobile crowdsensing systems is ambiguous and there is a variety of divergent trust models in different contexts, which is why challenges related to trust in mobile crowdsensing systems still remain unsolved [19]. Most common formalizations of trust and reputation frameworks that are designed for mobile crowdsensing systems adopt the Bayesian model, Gompertz function, vote-based mechanisms, majority voting, fuzzy logic models, or various other customized methods [17,29].

Huang et al. [23] proposed a system for evaluating the trustworthiness of participants’ contributions in mobile crowdsensing systems. They have proposed a trust and reputation framework that considers the fact that human users (ordinary citizens) carry sensors and produce information in mobile crowdsensing systems. The framework consists of a watchdog module and a reputation module. The watchdog module produces a rating of each user contribution using an outlier detection algorithm based on majority voting or robust average algorithm, depending on the type of input data. The ratings of the contributions are then used in the reputation module that computes a reputation score based on Gompertz function, which is well-suited to model dynamic human trust perception that is typical for mobile crowdsensing systems. In contrast, we use nonparametric statistics for comparison of participants’ attitudes. We evaluated performance of the method proposed by [23] and compared it with our proposed framework in Section 4.

Different trust and reputation frameworks that leverage online social networks to derive and model trust properties have been proposed [30,31,32,33,34,35,36], which differ in their approach to calculate trust values:

- Amintoosi and Kanhere [30] proposed an application agnostic framework to evaluate trust in social participatory sensing systems based on fuzzy logic and the PageRank algorithm. They leveraged existing online social networks and used friendship relations to identify and select suitable participants for certain tasks. They considered human involvement in mobile crowdsensing systems with the introduction of a subjective evaluation that enables the requester to indicate how much the contribution is compatible with his/her needs and expectations. It implies the trustworthiness of a contribution from the requester’s point of view. They utilized the PageRank algorithm to calculate a reputation score for each participant. They proposed another solution [31] that leverages social networks and proposes multi-hops, i.e., selection of friends of friends, in order to select the most appropriate and trustworthy participants for a task recruitment. Such multi-hop selection offers an access to a larger group of suitable participants, and increased the probability of accessing well-suited participants who are able to offer new perspectives and provide trustworthy contributions. In [31], the authors defined a suitability as an acceptable match between a participant’s trust factors and the task requirements, and assumed the requester may desire to add a subjective evaluation (as described above), which coincides with the assumptions used in our model.
- Another social network-based solution for assessing the trustworthiness of users was presented by Kantarci et al. [32]. They proposed Social Network-Aided Trustworthiness Assurance (SONATA) crowdsensing framework that is a recommendation-based approach that adopts vote-based trustworthiness analysis to identify malicious users. Later, they introduced “anchor” users in their model in order to avoid situations when malicious users collaborate, and they cast negative votes for the reputable users and positive votes for malicious users [33,34]. Anchor users are considered as fully trustworthy and fully capable of voting for the trustworthiness of other users in a mobile crowdsensing system.
- Nitti et al. [35] defined a subjective trust model for trustworthiness evaluation in the social Internet of Things, where each node computes the trustworthiness of its friends on the basis of its own experience, and on the opinion of the friends in common with the potential service provider. In their model, the trustworthiness of a node depends on who computes it, i.e., for  example, the  trustworthiness of node X as seen by node Y. They assume that trust is personal and asymmetric, since every participant has its own opinion about the other participants based on its personal experiences. The proposed model employs a feedback system and combines the credibility and centrality of the nodes to evaluate the trust level. Later, they extended a subjective model with an objective model [36], where a node’s trustworthiness is global for the entire network, and analyzed how the proposed subjective and objective models work with different dynamic behaviors of the nodes.

The solutions that leverage online social networks are close to our framework and idea behind it, as they compute trust value according to who requests it and not as a global value. In contrast to our work, the proposed trust frameworks require usage of social networks. Therefore, they are better suited to incentive users to contribute data or to perform other sensing tasks, since social friendship relations have positive effect on data contribution [30]. Furthermore, our proposed framework does not require an underlying social network and does not compare users based on their social relations or properties, such as common interests, eduction, etc. In the proposed framework, we compare users based on their trust evaluation dispositions. However, the proposed social network-based solutions and our proposed framework are complementary and could extend the functionalities of each other.

SACRM [37] is a Social Aware Crowdsourcing with Reputation Management model to select the well-suited participants for a specific sensing task and reward the participants adaptively, based on the quality of their sensing reports. The model considers social attributes, task delay and reputation for participants’ selection, whereas the trustworthiness of the sensing report is based on its similarity with other sensing reports, i.e., the amount of supports and conflicts it obtains from other sensing reports. The SACRM system is designed to maximize the crowdsourcing utility and to provide economical stimulations. In our work, a focus is on selecting well-suited participants to use their sensing contributions, but we have not addressed issues related to limited budget and platform utility. In contrast to SACRM, we proposed a high-level framework, which can be applied to broad spectre of mobile crowdsensing scenarios. Furthermore, we included trust differentiation in trust reasoning process, which is not considered in SACRM.

Yang et al. [38] proposed a framework for calculating reputation information and use it to select trustworthy participants and data. The model proposes indirect and direct measures of reputation, coupled with personal information in order to classify individuals as trustworthy or not. Direct reputation considers previous data quality records and participants’ past performance (objective information), whereas indirect reputation includes community trust and organizer’s trust (subjective information). According to the calculated trust value, they rank the participants and classify them as very trustworthy, trustworthy, untrustworthy, or very untrustworthy. The idea behind the proposed framework in similar to ours, however they presented the framework in a descriptive way and they did not include a method to evaluate the trustworthiness of users’ contributions. Therefore, it is impossible make a more precise comparison with our proposal.

A framework to define the most trusted participants for certain tasks based on geographic and temporal availability as well as participation habits was proposed by Reddy et al. [39]. They  proposed “typical behavior” of the participants that relates to their collected location traces. Similar approach was proposed by Kalidindi et al. [40]. They proposed a model that evaluates the trust of a participant considering personal and community opinion. The personal opinion is derived from the number of positive and negative interactions between participants. The assessment of the interaction (positive/negative) is defined by response time, time gap, familiarity, reciprocity and relevance. Relevance is assumed as a subjective parameter and presents the usefulness of the response. Personal and community opinions are then aggregated to derive a trust value of a participant. They assume that nodes (users) interact with each other, while our proposed framework does not. Proposed models [39,40] apply Beta distribution and custom mappings, which are suitable to measure the quality and quantity of contributions that are expected from users, but they lack the ability to capture dynamic and non-deterministic patterns as the result of an assortment of human behavior. In contrast to our work that presents an application agnostic trust framework, their solution is designed for specific scenarios and includes domain specific parameters such as response time, which may not be deployable in other mobile crowdsensing scenarios.

The described trust models have made some attempts to include human factors and elements of subjectivity in trust reasoning and are suitable for mobile crowdsensing systems. They include factors related to human involvement in mobile crowdsensing systems, i.e., personal opinions, subjective evaluations, personal needs and expectations, etc. However, in contrast to our work, they have not considered trust forming factors stemming from behavioral psychology such as irrationality and trust differentiation [21,22], which we have included in our proposed trust model. The referred solutions do not propose collecting two types of data for the same event as opposite to our proposed trust framework. Additionally, most of them are designed for a specific context or a particular use-case, in contrast to our trust framework, which is proposed in a general way and widely deployable for various mobile crowdsensing scenarios.

## 3. Proposed Conceptual Trust Framework and Method for Detecting Untrustworthy User Contributions

We propose a novel trust framework that formalizes trust-related factors in mobile crowdsensing systems. The objectives of the proposed framework are to identify key elements, properties and relations for managing trust in mobile crowdsensing environments and to define a novel method for trustworthiness evaluation of users’ contributions considering human aspects of trust reasoning, which is more efficient than the existing models in terms of error rate, minimal human involvement and wide applicability.

Trust formalization is an extension of our previous work on Qualitative Assessment Dynamics (QAD) [22,41,42]. The QAD considers trust as an expression of thinking and judgement processes originating in psychology [21]. It takes into account certain psychological facts and findings [22,43] and assumes the following trust forming factors:

1. User’s trust is driven by rational and irrational factors (rationality and irrationality).
2. Trust is a basis for a user’s actions and his/her ways of interaction with the environment (action binding).
3. Trust is not merely the product of an independent user’s thinking, but also influenced by the environment (feed-back dependence).
4. Trust is reflected in various forms due to various linguistic abilities of users to express trust and different perceptions of the ability of the evaluated entities (trust differentiation).
5. A user’s trust relation towards the object/subject is changing dynamically over time (time dynamics).

The previous extensions of the QAD include solutions to use sensors as a supportive element for evaluation of trust assessments and show its applicability for sensor-supported environments [43]. In  this paper, we present further extensions of the QAD that support trust management in human-centric mobile crowdsensing systems, where users contribute data explicitly and implicitly, as  well as evaluate the contributions. The proposed extensions of the QAD include introduction of events and the events’ trust values (assessments) that consist of a subjective and an objective part. Two parts of the assessments reflect the properties of the mobile crowdsensing paradigm that aims to exploit both: (1) pervasiveness of smart devices with advanced sensing capabilities; and  (2) willingness of users to contribute content. Based on that, we propose definitions of trust matrix, personal assessment vector, trust vector, trust value of the event, a user’s attitude and adjusted trust vectors. Using the proposed extensions, we define a method for data trustworthiness computation in mobile crowdsensing systems.

**Figure 1:**
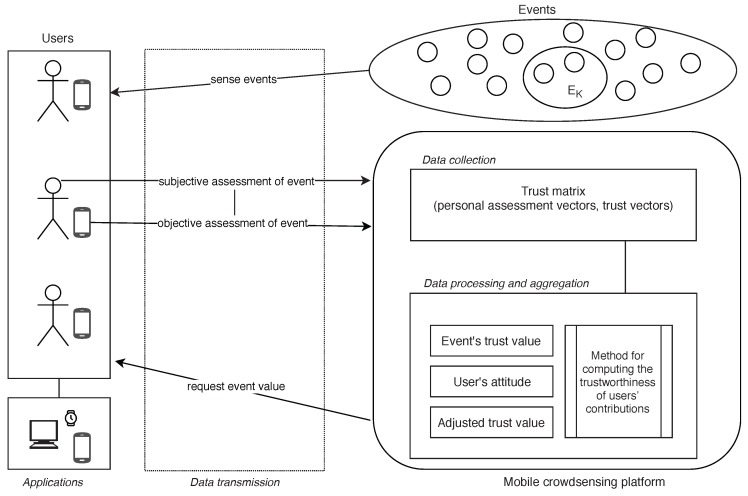
Trust framework architecture.

Figure 1 shows elements of the proposed trust framework, which are described in following subsections.

### 3.1. User

A mobile crowdsensing system consists of a set of users $U=\{u_{1},u_{2},u_{3},\ldots ,u_{n}\}$ that presents smartphone owners who contribute sensing data to the system. In mobile crowdsensing systems, users are often referred to as prosumers, since they act as both consumers and producers of crowdsensing data [44]. Another common notation is a participant. We use all three notations interchangeably.

### 3.2. Event

Users are present in an environment where different events from event set $E=\{e_{1},e_{2},e_{3},\ldots ,e_{m}\}$ can occur. Users collect data about the events and may contribute data to a mobile crowdsensing platform. The platform aggregates the data from multiple users and uses them to analyze the characteristics of the observed phenomenon (for example, to optimize public transport routes and timetables).

### 3.3. Event’s Assessment

A user captures two parts of data about a certain event—objective value (sensor readings, such as noise measurement) and/or subjective value (personal opinions or judgements, such as posted review) of the event. Acquiring objective and subjective values about sensing events requires different levels of user involvement and different sensing approaches:

1. Participatory sensing approach requires active involvement of individuals to contribute data, for  example, posting an opinion, filling a questionnaire, etc. related to an observed phenomenon. With the participatory sensing approach we capture subjective event values.
2. Opportunistic sensing is more autonomous and user involvement is minimal. For example, a smartphone can sample location continuously without explicit action from the user. The  opportunistic sensing approach is used to capture objective event values, obtained from the available sensors in a smartphone or other mobile device.

In our proposed framework, an event’s assessment consists of a subjective and an objective part. It is represented by $\omega_{i,j}=\left(\omega_{i,j}^{subj},\omega_{j}^{obj}\right)$, which denotes user $u_{i}$’s assessment of event $e_{j}$. The subjective part of the assessment $\omega_{i,j}^{subj}$ is contributed explicitly by the user with participatory sensing approach, while the objective part $\omega_{j}^{obj}$ is captured implicitly via opportunistic sensing approach. We refer to the subjective part of the event’s assessment as “subjective assessment” and to the objective part as “objective assessment” throughout the paper.

The subjective assessment $\omega_{i,j}^{subj}$ is taken from set $\Omega_{subj}=\left\{-2,-1,0,1,2\right\}$, where the numbers symbolize distrusted, partially distrusted, undecided, partially trusted and trusted values for the event description. Assessment values could be given in other textual representation, such as strongly disagree, disagree, neutral, agree and strongly agree, depending on an application-specific context and content. Independently of the selected formulation, our model proposes a qualitative and ordinal data set of assessment values, since such data set is understood and manipulated by humans easily. If a user has not assessed an event yet, then the subjective part of the event assessment is not defined and denoted with symbol “/”.

The objective assessment $\omega_{j}^{obj}$ has a real number value on the interval $\Omega_{obj}\in \left[0,1\right]$. It is used for comparison of events and their classification. We define a group of same events as set $E_{k}=\left\{e_{k}\in E|\omega_{k}^{obj}-\tau \leq \omega_{k}^{obj}\leq \omega_{k}^{obj}+\tau \right\}$, where τ denotes events classification threshold. For example, events that happen at the same time and at the same geographic location would have the same objective value in a mobile crowdsensing application that collects data about traffic conditions. In real-case scenarios, it  is highly unlikely that two events have exactly the same objective value. Therefore, throughout the paper we use the terminology same events, which describes that the events are in the same group. Similarly, a notion “an event” can refer to an event or a group of same events, interchangeably.

Event assessments are stored in trust matrix M. A general form of the trust matrix is as follows:

$$M=\left[\begin{array}{cccc} \left(\omega_{1,1}^{subj},\omega_{1}^{obj}\right) & \left(\omega_{1,2}^{subj},\omega_{2}^{obj}\right) & \ldots & \left(\omega_{1,m}^{subj},\omega_{m}^{obj}\right)\\ \left(\omega_{2,1}^{subj},\omega_{1}^{obj}\right) & \left(\omega_{2,2}^{subj},\omega_{2}^{obj}\right) & \ldots & \left(\omega_{2,m}^{subj},\omega_{m}^{obj}\right)\\ \vdots & \vdots & \ddots & \vdots \\ \left(\omega_{n,1}^{subj},\omega_{1}^{obj}\right) & \left(\omega_{n,2}^{subj},\omega_{2}^{obj}\right) & \ldots & \left(\omega_{n,m}^{subj},\omega_{m}^{obj}\right) \end{array}\right].$$

Row *k* in the trust matrix M represents user $u_{k}$’s personal assessment vector. It contains user $u_{k}$’s assessments of events and is denoted as $M_{k,m}=\left\{\left(\omega_{k,1}^{subj},\omega_{1}^{obj}\right),\left(\omega_{k,2}^{subj},\omega_{2}^{obj}\right)\ldots \left(\omega_{k,m}^{subj},\omega_{m}^{obj}\right)\right\}$. Furthermore, $\underline{M_{k,m_{k}}}=\left\{\left(\omega_{k,1}^{subj},\omega_{1}^{obj}\right),\left(\omega_{k,2}^{subj},\omega_{2}^{obj}\right)\ldots \left(\omega_{k,m_{k}}^{subj},\omega_{m_{k}}^{obj}\right)\right\}$ denotes user $u_{k}$’s assessments of the events where undefined values “/” are omitted. Furthermore, notations $M_{k,m}^{subj}$ and $\underline{M_{k,m_{k}}^{subj}}$ denote vectors that contain only subjective parts of the assessments, whereas $M_{k,m}^{obj}$ and $\underline{M_{k,m_{k}}^{obj}}$ mark vectors that contain only objective parts of the assessments.

### 3.4. Event’s Trust Value

In the trust matrix M, column *k* represents trust vector about event $e_{k}$. It holds assessments (given by users) about particular event $e_{k}$ and is denoted as $M_{n,k}=\left\{\left(\omega_{1,k}^{subj},\omega_{k}^{obj}\right),\left(\omega_{2,k}^{subj},\omega_{k}^{obj}\right)\ldots \left(\omega_{n,k}^{subj},\omega_{k}^{obj}\right)\right\}$. We denote a trust vector with omitted “/” values with $\underline{M_{n_{k},k}}=\left\{\left(\omega_{1,k}^{subj},\omega_{k}^{obj}\right),\left(\omega_{2,k}^{subj},\omega_{k}^{obj}\right)\ldots \left(\omega_{n_{k},k}^{subj},\omega_{k}^{obj}\right)\right\}$. Notations $M_{n,k}^{subj}$ and $\underline{M_{n_{k},k}^{subj}}$ denote vectors that contain only subjective parts of the assessments, and $M_{n,k}^{obj}$ and $\underline{M_{n_{k},k}^{obj}}$ represent vectors that contain only objective parts of the assessments.

The trust value of an event $e_{k}$ is defined as follows:

(1) $$\rho_{e_{k}}=\frac{1}{n_{k}}\sum_{\omega_{i,k}^{subj}\in \underline{M_{n_{k},k}^{subj}}}\omega_{i,k}^{subj}.$$

### 3.5. Users’ Attitudes

The success of mobile crowdsensing systems depends on a large number of participating users. The openness of the mobile crowdsensing paradigm allows anyone to contribute the data, including malicious users. Malicious users post erroneous and malicious data, inadvertently or deliberately. In  the first case, we assume that users are inexperienced or careless in generating and reporting event assessments to the platform, which results in an assessment of an event that has a different value than the actual assessment of the event. In the latter case, we assume that the users report false values deliberately in order to achieve a certain benefit, such as gaining a reward for large numbers of contributions, or to decrease the trust value of a competitive service. A mobile crowdsensing platform aggregates contributed data. If users contribute data that do not reflect true values, then the data aggregation results are useless.

For this reason, it is essential that the trust framework include mechanisms to evaluate the trustworthiness of the user contribution in order to provide users with results that are useful to them. With the proper method, the mobile crowdsensing platform is able to provide more reliable information that can be used in further analysis. Our proposed method takes into account different user behaviors that also affect the quality and trustworthiness of reported data.

We derive the characteristics of a user’s assessment disposition from their subjective assessments of the events obtained from their personal assessment vector. In our model, a user’s attitude is an estimation of the true underlying cumulative distribution function of the subjective assessments in their personal assessment vector, obtained with an empirical cumulative distribution function. User $u_{i}$’s attitude $F_{i,m_{i}}\left(\omega^{subj}\right)$ is represented with an empirical cumulative distribution function of a data set $\underline{M_{i,m_{i}}}=\left(\omega_{i,1}^{subj},\omega_{i,2}^{subj},\ldots \omega_{i,m_{i}}^{subj}\right)$ and is obtained as:

(2) $$F_{i,m_{i}}\left(\omega^{subj}\right)=\frac{1}{m}\sum_{j=1}^{m_{i}}\left(I\left(\omega_{i,j}^{subj}\right)\leq \omega \right),$$

where $\left(I\left(\omega_{i,j}^{subj}\right)\leq \omega^{subj}\right)$ is the indicator function equal to 1 if $\left(\omega_{i,j}^{subj}\right)\leq \omega^{subj}$ and equal to 0 otherwise.

### 3.6. Event’s Adjusted Trust Value

In our proposed model, users with different behavioral patterns are not classified in distinct groups, but each can be treated independently with regard to their attitudes. As such, we can compare each user with other users separately and find similar users, which means users with similar attitudes, as defined above. The comparison of attitudes means comparison of two independent subjective event assessment distributions, which can be performed with different statistical tests. A  mobile crowdsensing platform typically has no knowledge about parameters that describe users’ event assessment distributions. For this reason, we use nonparametric statistics. The nonparametric statistical tests make no assumption about the population distribution or sample size, which make them suitable for attitude comparison. We use the Kolmogorov-Smirnov (KS) test for two samples, which is more appropriate and more powerful than the other comparable nonparametric tests, i.e., the Wilcoxon signed-rank test, the Mann-Whitney test, the Kruskal-Wallis test. The two-sample Kolmogorov-Smirnov test has less power to detect a shift in the median, but more power to detect changes in the shape of the distributions. Therefore, it is entirely appropriate for a comparison of assessment distributions, i.e., the comparison of the users’ attitudes, and powerful enough to detect changes in the shape of distribution that occurs due to false data posted by malicious users.

We compute the similarity between the users by applying the two-sample KS test. Let $F_{i,m_{i}}\left(\omega^{subj}\right)$ and $F_{j,m_{j}}\left(\omega^{subj}\right)$ be the attitudes of users $u_{i}$ and $u_{j}$. The similarity between the users $u_{i}$ and $u_{j}$ is defined as:

(3) $$sim\left(u_{i},u_{j}\right)=1-sup_{\omega^{subj}}\left|F_{i,m_{i}}\left(\omega^{subj}\right)-F_{j,m_{j}}\left(\omega^{subj}\right)\right|,$$

where $sim\left(u_{i},u_{j}\right)=\left\{x\in R\;|\;0\leq x\leq 1\right\}$.

Furthermore, we derive a trust vector about event $e_{k}$, adjusted to user $u_{i}$’s perspective. An ordered trust vector is defined as:

(4) $${\overset{\leftarrow }{\underline{M}}}_{[n_{k}],k}^{i}=\left[\omega_{[1],k}^{subj},\omega_{[2],k}^{subj},\ldots \omega_{[n_{k}],k}^{subj}\right],\forall \left[p\right]<\left[r\right]:sim\left(u_{i},u_{\left[p\right]}\right)\geq sim\left(u_{i},u_{\left[r\right]}\right)$$

We define a user $u_{i}$-adjusted trust vector about the event $e_{k}$ as follows:

(5) $${\overset{\leftarrow }{\underline{M}}}_{[n_{s}],k}^{i}=\left[\omega_{[1],k}^{subj},\omega_{[2],k}^{subj},\ldots \omega_{[n_{s}],k}^{subj}\right],\forall \omega_{[j],k}^{subj}:\left[j\right]\geq \left[simTh\right].$$

A user $u_{i}$-adjusted trust vector contains assessments about a certain event, where it includes only those assessments contributed by the other users that are sufficiently similar to $u_{i}$ and do not post false or unsuitable data. An adjusted trust vector contains simTh best fitting values, according to similarities with the other users in the mobile crowdsensing system. A user $u_{i}$-adjusted trust value of the event $e_{k}$ is derived as:

(6) $$\rho_{e_{k}}^{i}\left(\overset{\leftarrow }{{\underline{M}}_{[n_{s}],k}^{i}}\right)=\frac{1}{\left[n_{s}\right]}\sum_{\omega_{i,k}^{subj}\in {\overset{\leftarrow }{\underline{M}}}_{[n_{s}],k}^{i}}\omega_{i,k}^{subj}.$$

### 3.7. Method for Computing the Trustworthiness of Users’ Contributions

Based on the proposed formalization, we define the method that filters users’ contributions that are recognized as untrustworthy. The purpose of the proposed method is to determine the trustworthy assessments of a particular (or user unknown) event given by other participants, and to compute the trust value of a reported event. Adjusted trust value of an event allows each individual user to make an unbiased comparison and make decisions. Algorithm 1 describes the proposed method.

The proposed method for computing the trustworthiness of users’ contributions mitigates their possible misinterpretations. The method does not require the explicit involvement of the participants, except sharing assessment of the events. These are stored in a trust matrix M, held by the mobile crowdsensing platform that collects user contributions. A user’s sharing of subjective values about the events requires their active involvement on the mobile application level, while contributing objective values of the events uses the opportunistic sensing approach with no or minimal user involvement.

**Algorithm 1** Method for computing a trust value of an event  **Input:** user $u_{i}$, event $e_{k}$, simTh, evtnSim  **Output:**
$\rho_{e_{k}}^{i}$: trust value of $e_{k}$

 1:A←{} 2:Derive attitude of user $u_{i}$ // Equation (2) 3:Find $e_{K}\in E_{K}:\omega_{K}^{obj}=\omega_{k}^{obj}\pm evtnSim$ 4:**for** each $u_{j}$ **do** 5:  **if** $\omega_{j,K}^{subj}\neq$ “/” **then** 6:  Derive attitude of user $u_{j}$ // Equation (2) 7:  Put $\omega_{j,k}^{subj}$ to $\underline{M_{n_{k},k}^{subj}}$ 8:  Compute $sim(u_{i},u_{j})$ // Equation (3) 9:  **end if**10:**end for**11:Sort $\underline{M_{n_{k},k}^{subj}}$ // Equation (4)12:Set j=113:**while**j≥simTh**do**14:  Put $\omega_{[j],k}^{i}$ to a A // Equation (5)15:  
j=j+1
16:**end while**17:Compute $\rho_{e_{k}}^{i}\left(A\right)$ // Equation (6)


## 4. Evaluation

In the previous section we described our conceptual trust framework for mobile crowdsensing systems and a novel method to evaluate the trustworthiness of participants’ contributions. In this section, we present the results of the experimental evaluation of the proposed framework and further discuss its properties.

### 4.1. Experimental Evaluation

In recent years, several research facilities and experimental frameworks have been developed in order to facilitate real-world mobile crowdsensing scenarios and to collect massive amounts of useful data [45,46,47]. Furthermore, more advanced solutions were proposed that integrate various tools into a large-scale platform and enable consolidation of data from different sources [48,49]. These efforts produced real-world datasets that are available for further processing and analysis [50,51]. However, the available datasets contain data in a form that is not applicable for an evaluation of the conceptual trust framework that is proposed in this work. The proposed framework differs from existing trust management solutions in that it proposes to collect two types of the data (i.e., directly expressed assessment by humans and sensing reports captured by smart device sensors) describing the same event, which are related to each other but are used separately in further data analysis. The available datasets include data collected through realized experiments, such as mobility traces of buses, social interaction and propinquity data, accelerometer samples, social networking data, etc., but do not contain assessments expressed by users.

Therefore, we designed a simulation tool that implements scenario that is presented later in this section. The simulation tool implements three different methods for an event’s trust value computation:

- Firstly, the simulation tool implements the proposed method, described in Section 3 (denoted as Subj.).
- Secondly, a method proposed by Huang et al. [23] is implemented in the simulation tool (denoted as Gomp.). In their proposal [23], trust and reputation management system is made up of two components—(i) watchdog module and (ii) reputation module. The watchdog module implements the majority-voting algorithm in order to detect outliers and evaluates trustworthiness of each contribution. In this step, candidates of events’ assessment values’ are defined, which act as input to the reputation module. The reputation module builds a long-term view of the trustworthiness of contributed events’ assessments. It applies Gompertz function in order to define the trust value of the assessments that are recognized as solution candidates in the watchdog module. In each configuration with Huang’s method we used the following parameter values, a=1, b=−2.5, c=−0.85 and threshold=0.5, which are the same values as used in the authors’ evaluation of the method [23].
- Thirdly, to provide a baseline for comparing methods, simulation tool also implements a calculation of a trust value of an event without considering the trustworthiness of the data (denoted as W/O).
  In this case, the event’s trust value is computed as an average value of all subjective assessments of the event previously reported by users, as defined in Equation (1).

Although several solutions that address trustworthiness of contributed data in mobile crowdsensing systems have been proposed in the literature and research has been performed regarding trust and reputation systems in mobile crowdsensing systems people-centric factors and factors related to dynamism in human behavior have not been formalized. Nevertheless, [23] proposed a reputation system that uses Gompertz function, whose mathematical construct is well-suited to model the asymmetry in managing reputation for people-centric devices. Their method is suitable to handle differences and transitions in a user’s trust behavior. Therefore, we have compared our method with Huang’s method, which, to our best knowledge is closest to our work and suitable for a comparison.

The simulation tool has been developed in Java JDK 8 using Eclipse IDE. The simulations were conducted on MacOS 10.13 with 3,1 GHz Intel Core i5 processor and 16 GB RAM and carried out in a controlled environment, with all background processes stopped. The simulation tool has been extensively tested, verified and validated to assure that the simulated results are conformed with each method. To achieve this, we have verified the output of each simulation step and compared the results using manual calculations and calculations computed with Mathematica tool. We have also implemented automated tests using JUnit.

The implemented simulation tool allows to set parameters, such as number and type of events, number and personality types of users, number of malicious users, Gompertz function parameters, threshold values, etc. Setting the parameters to different values allows to execute the scenario anticipating different circumstances. We have set parameters for each configuration, as follows.

The simulation environment consists of n=100 users and m=30 events. At the beginning, all events are not assessed yet, i.e., $\omega_{i,j}^{subj}=$ “/”, ∀i,j. An event has an objective value that remains unchanged during the simulations. Events have defined objective values with uniform distribution, such that $\omega_{1}^{obj}=\frac{1}{m}$, $\omega_{2}^{obj}=\frac{2}{m}$, $\omega_{3}^{obj}=\frac{3}{m}$, etc.

We define six basic personality types in order to simulate different human behaviors. Personality types are elements of set Ψ={⇑,⇓,∼,↔,↑,↓}, where the symbols denote optimistic, pessimistic, centralistic, opportunistic, moderately optimistic and moderately pessimistic personality types, respectively. The personality type is a function ψ∈Ψ that defines how a user with a certain personality type evaluates an event. The functions of the particular personality types are defined as follows:

$${⇑}_{i}:\omega_{i,j}^{subj}\left(\omega_{i}^{obj}\right)=\left\{\begin{array}{cc} 0; & \omega_{i}^{obj}<0.25\\ 1; & 0.25\leq \omega_{i}^{obj}<0.5\\ 2; & 0.5\leq \omega_{i}^{obj} \end{array}\right.$$

$${⇓}_{i}:\omega_{i,j}^{subj}\left(\omega_{i}^{obj}\right)=\left\{\begin{array}{cc} -2; & \omega_{i}^{obj}<0.5\\ -1; & 0.5\leq \omega_{i}^{obj}<0.75\\ 0; & 0.75\leq \omega_{i}^{obj} \end{array}\right.$$

$${∼}_{i}:\omega_{i,j}^{subj}\left(\omega_{i}^{obj}\right)=\left\{\begin{array}{cc} -2; & \omega_{i}^{obj}<0.2\\ -1; & 0.2\leq \omega_{i}^{obj}<0.4\\ 0; & 0.4\leq \omega_{i}^{obj}<0.6\\ 1; & 0.6\leq \omega_{i}^{obj}<0.8\\ 2; & 0.8\leq \omega_{i}^{obj} \end{array}\right.$$

$${↔}_{i}:\omega_{i,j}^{subj}\left(\omega_{i}^{obj}\right)=\left\{\begin{array}{cc} -2; & \omega_{i}^{obj}<0.25\\ -1; & 0.25\leq \omega_{i}^{obj}<0.5\\ 1; & 0.5\leq \omega_{i}^{obj}<0.75\\ 2; & 0.75\leq \omega_{i}^{obj} \end{array}\right.$$

$${↑}_{i}:\omega_{i,j}^{subj}\left(\omega_{i}^{obj}\right)=\left\{\begin{array}{cc} -2; & \omega_{i}^{obj}<0.1\\ -1; & 0.1\leq \omega_{i}^{obj}<0.3\\ 0; & 0.3\leq \omega_{i}^{obj}<0.5\\ 1; & 0.5\leq \omega_{i}^{obj}<0.7\\ 2; & 0.7\leq \omega_{i}^{obj} \end{array}\right.$$

$${↓}_{i}:\omega_{i,j}^{subj}\left(\omega_{i}^{obj}\right)=\left\{\begin{array}{cc} -2; & \omega_{i}^{obj}<0.3\\ -1; & 0.3\leq \omega_{i}^{obj}<0.5\\ 0; & 0.5\leq \omega_{i}^{obj}<0.7\\ 1; & 0.7\leq \omega_{i}^{obj}<0.9\\ 2; & 0.9\leq \omega_{i}^{obj} \end{array}\right.$$

Users with different personality types perceive and subjectively evaluate events with the same objective value differently. For example, an event $e_{1}$ has an objective value $\omega_{1}^{obj}=0.65$. A user with optimistic personality type subjectively evaluates the event as trusted, a user with pessimistic personality type as partially distrusted, a user with centralistic, opportunistic or moderate optimistic personality type as partially trusted, while a user with moderate pessimistic personality type evaluates the trust value of the event as undecided. The subjective evaluations conflict due to different personality types of users and do not impose that one of them reports true assessment of an event, while other are malicious and report false trust assessments of an event.

### 4.2. Simulation Scenario

We simulate a mobile crowdsensing environment where different kinds of events happen and participants contribute the assessments of the events to the platform using a simulation tool. In the simulation tool, the platform stores both subjective and objective values of the events and uses those values to evaluate event trust value. In each time step of the simulation, we execute the following simulated scenario:

1. User $u_{S}$ requests a platform for a trust value of an event $e_{R}$.
2. The platform computes the trust value of the event $e_{R}$ based on users’ contributions considering their trustworthiness. The platform computes the trustworthiness level of the (previously) contributed data using:
  - Our proposed method (Subj.); or
  - Huang’s method (Gomp.); or
  - Without trustworthiness computation (W/O).
3. The event $e_{R}$ happens and user $u_{S}$ perceives it.
4. The objective value of the event $e_{R}$ is stored by the platform (without user involvement).
5. User $u_{S}$ assesses the event.
6. User $u_{S}$ sends a subjective assessment to the platform.
  - A good user sends a true value.
  - A malicious user sends a false value.
7. *Evaluation step:* Comparison of the computed trust value of the event and actual assessment of the event.

In the first step of the simulated scenario, a user $u_{S}$ requests a platform for a trust value of an event $e_{R}$. We use the phrases “same events” or “an event” throughout the paper, which both refer to a group of same events, as defined in Section 3. In the executed simulations, the events classification threshold is τ=0.05.

Then, we compute the trustworthiness of the user contributions in three different ways (using Subj., Gomp. or W/O) in order to compare results and to evaluate the effectiveness of our proposed approach. In Steps 3–6 of the simulated scenario, the user perceives and assesses the event. The subjective assessment of an event is defined with the personality type of the user, which does not change during the simulation. The above defined personality types are used to calculate the subjective value of an event, regardless of which method to calculate the events’ trust value is used.

However, we assume that a user may contribute a false assessment of an event to the platform (Step 6 of the simulated scenario). In the simulations we define a good user as a user who always contributes correct data. Furthermore, we define a malicious user as a user who reports false data. Malicious users may report false data inadvertently or deliberately. We do not differentiate between them, since both contribute untrustworthy data. In this work and simulations performed, a malicious user reports an assessment that has lower value, such that $\omega_{M,j}^{subj}=max\left(\bar{\omega_{M,j}^{subj}}-2,2\right)$, where $\bar{\omega_{M,j}^{subj}}$ denotes a real subjective value of an event as perceived in Step 3.

Step 7 of the simulated scenario is an additional step to evaluate the performance of the selected method for data trustworthiness computation. After each transaction, i.e., a user’s request for an event trust value, a platform’s response and an actual assessment of the event, we check if the platform provided the correct trust value (in Step 2 of the simulated scenario). A correct value is considered to be the same value as how the user evaluates the event (in Step 5 of the simulated scenario).

**Figure 2:**
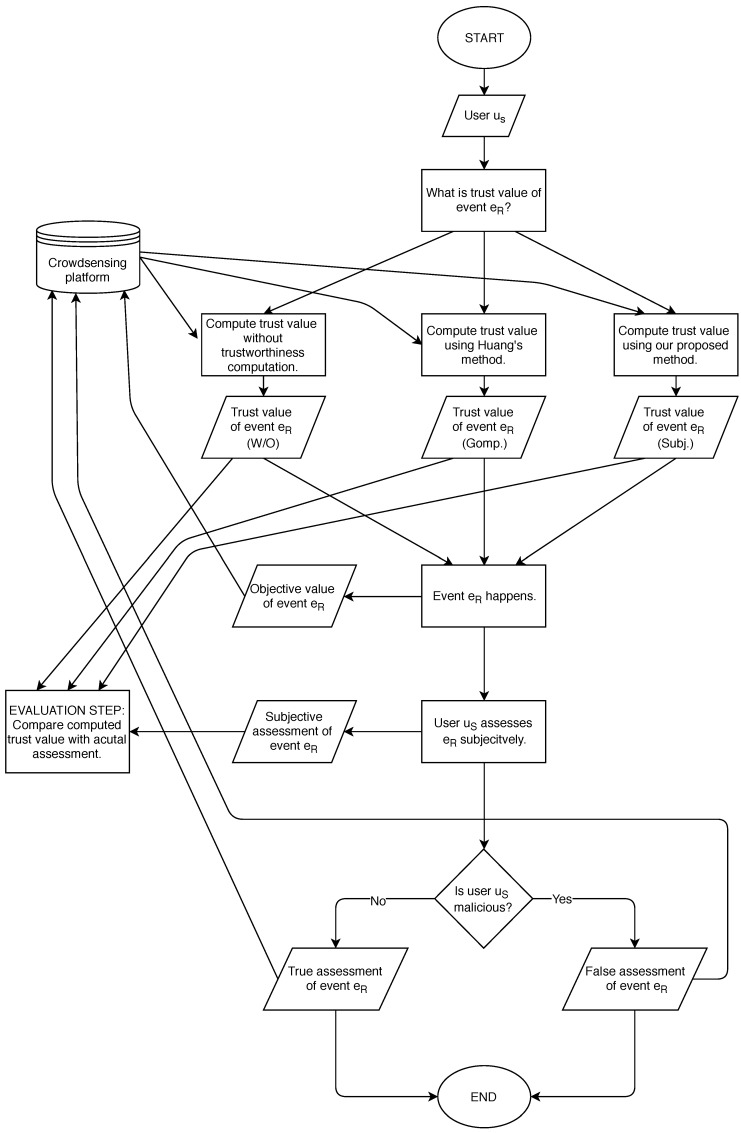
Algorithm chart for simulated scenario.

In each time step of the simulation, the above scenario (Steps 1–7) executes with a random user and a random event. We describe the simulated scenario with an algorithm chart in Figure 2.

We have run simulations for 1000 steps. In real case scenarios users do not have knowledge about all events (otherwise, there would be no need for trust management systems), meaning the trust matrix is sparse. In 1000 steps we collect ~33% of possible event assessments in a configuration with n=100 users and m=30 events, while other elements (~67%) in the trust matrix are undefined (“/”).

We calculated the error rate for each method. The error rate is the number of incorrect values over the total number of all computed values. The event’s value is computed upon the user’s request. The method computes the event’s trust value using previous users’ contributions about events that have already happened. The evaluated methods differ in how they define the trustworthiness of the users’ contributions and how they use them for the calculation of the event’s trust value. A low error rate indicates that a method is effective in computing an event’s trust value.

We carried out two series of experiments in order to evaluate the efficiency of our proposed method compared to Huang’s method and method without trustworthiness computation. First, we evaluated the methods depending on the number of malicious participants in a simulated mobile crowdsensing environment. Second, we compared them in environments with different distributions of users’ personality types.

### 4.3. Efficiency of Methods Depending on the Number of Malicious Users

In the first series of experiments, we evaluated the efficiency of the methods depending on the number of malicious users in the community. We ran the simulations with n = 100 users, with a uniform personality type distribution, i.e., there are 16.67% optimistic users, 16.67% moderately optimistic users, 16.67% users with a centralistic personality type, 16.67% moderately pessimistic users, 16.67% pessimistic users and 16.67% users with an opportunistic personality type.

We designed the simulation configurations with different percentages of malicious participants in the environment: 0% (only good users), 10%, 20%, 30%, 40% and 50%. We assume that a malicious user acts individually and reports false values for 30% of events. For the other 70% of events the malicious user reports true values. Every malicious user has his/her own “targets”, i.e., events for which the false values are reported.

We do not consider colluding attack scenarios, where a malicious user colludes with other users to create a false evaluation of a particular event, since the possibility of realizing such a scenario in real cases is small. Namely, mobile crowdsensing systems have some unique features. Typically, they involve location dependency, temporal continuity and participation in micro-tasks [52]. Mobile crowdsensing systems are based on and designed for micro-tasks, which results in micro-payments or micro-rewards [39,53,54] or no rewards at all [17]. The organization of a group attack requires effort, implying that the execution of a collusion attack may be uneconomic and unrewarding for their long-term reputation. Additionally, sensing reports in mobile crowdsensing systems typically include spatio-temporal constraints, which makes it difficult to organize and carry out a collusion attack. Therefore, we have not considered colluding attacks in the simulations.

**Figure 3:**
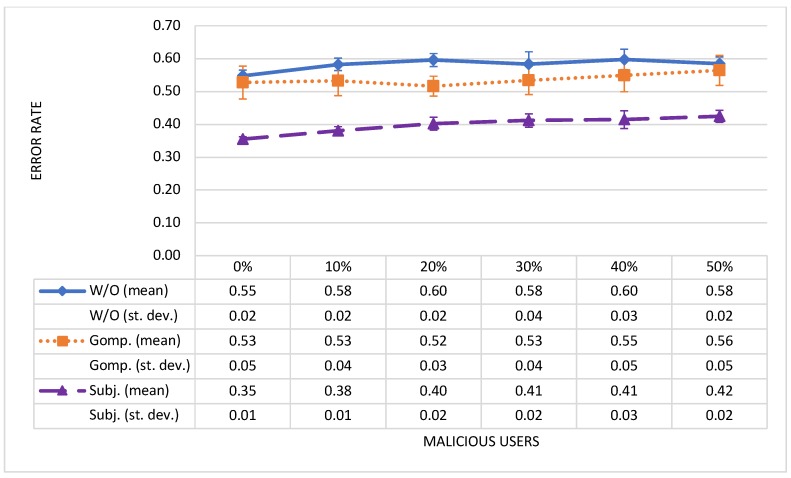
Mean value and Standard Deviation for error rate achieved with different methods for simulation configurations with different percentages of malicious users.

Each configuration with a different percentage of malicious users in the environment was executed for 1000 time-steps. We repeated each configuration 100 times and computed the average error rate for each simulation configuration with different percentage of malicious users, and executed with every method (W/O, Gomp. and Subj.). The mean value and Standard Deviation for the error rate, calculated after 100 repetitions of each simulation configuration, are presented in Figure 3.

Error rate increases with an increased number of malicious users in the community. In all configurations with different percentages of malicious users, the error rate achieved with our method is lower than the error rate achieved with Huang’s method or the method without data trustworthiness computation. The error rates vary from 0.35 (configuration with good users only) to 0.42 (configuration with 50% of malicious users) for our method, and from 0.53 (configuration with good users only) to 0.56 (configuration with 50% of malicious users) for Huang’s method. When computing the reputation of an event without considering the trustworthiness of users’ contributions, the error rate varies between 0.55 and 0.60. With the proposed method we improved the error rate by 32.6%, 28.5%, 22.1%, 22.9%, 24.6% and 24.8% over Huang’s method for configurations with 0%, 10%, 20%, 30%, 40% and 50% of malicious users, respectively. Compared to calculating the reputation of the events without considering data trustworthiness level, our proposed method improved error rate by 35.2%, 34.7%, 32.5%, 29.5%, 30.7% and 27.4% in configurations with 0%, 10%, 20%, 30%, 40% and 50% of malicious users, respectively.

Huang’s method calculates the reputation of users based on past experience with this user, depending on how trusted his/her contributions were in the previous interactions. The user’s reputation has a global value, which means that this value is the same for all users. For example, if user $u_{X}$ asks for an event’s $e_{Q}$ trust value (Step 1 in the simulated scenario), the calculation of the event’s $e_{Q}$ trust value is the same as if user $u_{Y}$ would ask for this event assessment. Similarly, the trust value of the event is also global in a method without data trustworthiness consideration. In this case, it is considered that all participants previously contributed assessments that are equally trustworthy.

Our method handles it in a different way. Namely, our method does not calculate a global (general) trust value, but a trust value that is adjusted according to who it is computed for. For example, user $u_{P}$ is an extreme pessimist who evaluates events with low values, although they are objectively good. In this case, the trust value of the event $e_{Q}$ is not automatically low, because the user $u_{P}$’s assessments are not in accordance with the majority. Conversely, if a requesting user $u_{S}$ (in Step 1 in the simulated scenario) is also inclined to evaluate events with low values, the trust value of the event $e_{Q}$ is comparatively high. Our model suggests that user $u_{S}$ perceives the considered event in the same way as user $u_{P}$ and evaluates it with the same value as user $u_{P}$. The calculated and the real assessment of the considered event is, thus, very likely the same, which results in a lower error rate. In an alternative case where user $u_{S}$ is inclined to different event assessments than user $u_{P}$ (for example, if the user is an optimist), data contributions are considered as untrustworthy for user $u_{P}$ and are not used in the computation of the trust value of the event in this case.

### 4.4. Efficiency of Methods Depending on Distribution of Personality Types

In the second set of experiments we assessed the performance of our proposed method depending on the distribution of participants with different personality types. We ran simulations with n = 100 users with the following distributions of personality types, as given in Table 1.

In the uniform distribution configuration there is the same number of users with each defined personality type. In the bipolar distribution configuration there are only two types of users—moderately pessimistic and moderately optimistic users. The first ones tend to assess events with higher values, whereas the others tend to provide lower assessments of the events. However, their assessments deviate in a positive/negative direction only to a small extent in comparison with the centralistic personality type. In the configuration with moderate distribution of personality types, there are the same number of centralistic, moderate optimistic and moderate pessimistic users. All three types assess events relatively evenly, with the assessments of the moderate optimistic users somewhat diverging upwards and the assessments of the moderate pessimistic users diverging slightly in a negative direction. Therefore, assessments of the same event, calculated by each type of user, are close. On the contrary, assessments of the same event differ more in configuration with extreme distribution of personality types. In the extreme distribution configuration, the simulated community consists of users with optimistic, pessimistic and centralistic personality types. The assessments computed by centralistic users are evenly distributed (assuming events have an even distribution of objective values). The assessments of optimistic and pessimistic users are extreme, compared to those computed by centralistic users. The optimistic users tend to give assessments with extremely high values compared to the majority of the assessments; and the pessimistic users tend to assess the event with extremely low values. Therefore, in the configuration with the extreme distribution of personality types, the assessments of the same event, calculated by each type of user, differ considerably.

In each configuration there are 20% of malicious users who report false assessments of events. The same as in the previous set of experiments, we assume that a malicious agent acts individually and reports false values for 30% of events.

**Figure 4:**
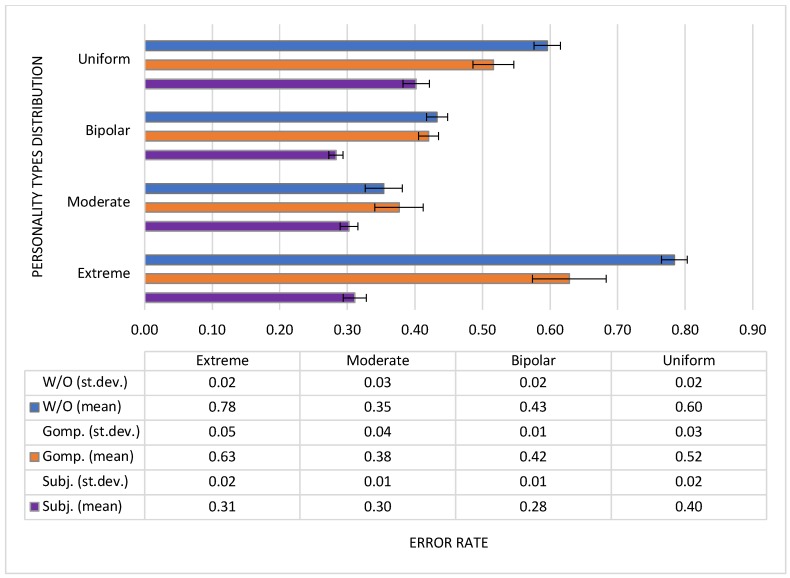
Mean value and Standard Deviation for error rate achieved with different methods for simulation configurations with different distributions of personality types.

We repeated each configuration 100 times and computed the average error rate for each simulation configuration. The mean value and Standard Deviation for the error rate calculated after 100 repetitions of each simulation configuration are presented in Figure 4.

In all configurations, our method achieved the lowest error rate that was improved by 22.1%, 32.7%, 19.6% and 50.5% over Huang’s method for configurations with Uniform, Bipolar, Moderate and Extreme distribution, respectively. Compared to the baseline method, which does not consider data trustworthiness, we improved the error rate by 32.5%, 34.6%, 14.5% and 60.3% in Uniform, Bipolar, Moderate and Extreme configurations, respectively.

In Extreme configuration, there are optimistic, centralistic and pessimistic participants in the simulated mobile crowdsensing environment. In that case, computing a trust value of the event as average (method W/O) is not suitable for a requesting user. For example, if a requesting user is a pessimist, then the computed trust value of the event is lower than the optimist user would assess it. The computed and the real value differ, resulting in a high error rate. Huang’s method performs well in Extreme configuration when a requesting user has a centralistic type of personality, since majority opinion converges to centralistic values. If the requesting user is pessimistic, then the solution candidates (i.e., assessments of the event) have, on average, higher values than the pessimistic user’s opinion. Similarly, if the requesting user is optimistic, then the majority-based opinion is lower.

Our method takes in the calculation only assessments of the event that are posted by users with similar attitudes. In Uniform configuration that consists of users with all possible types of personality, who evaluate events in different ways, a group of users with the same (similar) attitude is small. Therefore, if the user is a moderate optimist, the best fitting event assessment calculation would take only assessments reported by other moderate optimists from the community—assuming they are not malicious, since, in this case, the trust attitude does not match the trust attitude of the requesting user. Because, in this case, the group of moderate optimists is small, data contributions of optimists and centralists are taken into account, since they fall into the set of the most suitable users. The events’ assessment values are more dispersed in this case, which leads to the differences between the calculated and the true values and, consequently, to a higher error rate.

### 4.5. Discussion and Use Cases

The proposed framework assumes that an application uses both opportunistic and participatory sensing approaches for collecting data. Collecting two types of data for the same event has certain advantages that were already recognized. It exploits both high-performance sensors that are integrated into smartphones and smartphone owners’ intelligence in order to gain a better knowledge of the characteristics of the observed phenomena. Similar approaches to divide the captured sensing data in different parts have been used [23,55]. Wang et al. [55] refer to data captured via participatory sensing approach as “payload data”, which are obtained next to the contextual objective data and could be of any format, e.g., text, voice, picture, video. Advantages of subjective event assessment include human perception and understanding of the events in a specific contexts. Additionally, human users can handle large amounts of data and derive semantically complex information that complement the measurements of hardware sensors significantly. In the same work [55], an objective event value is referred to as “provenance data” and includes meta-data that describe the origin of the report and other contextual factors. It is assumed to be generated automatically by smart devices that users possess. Huang et al. [23] do not introduce special notation for different parts of the sensed data, but also tag reported data with additional information, such as time and location.

The proposed trust framework, which includes the method for detecting untrustworthy user contributions is general and widely applicable to different kinds of mobile crowdsensing applications. It can be used as an addition to existing mobile crowdsensing applications in order to improve the interpretation of contributed data and their trustworthiness evaluation. Application of the proposed framework for a specific domain requires that collected subjective and objective values of the sensed data are mapped in the proposed domains, i.e., $\Omega_{subj}=\left\{-2,-1,0,1,2\right\}$ and $\Omega_{obj}\in \left[0,1\right]$. The mapping is context-specific, whereby the proper level of data granularity should be applied to receive meaningful data contributions. For example, an event value $\omega_{j}^{obj}\in \left[0,1\right]$ can denote noise level between 30 dB and 100 dB in a room noise monitoring application, where the mapping is not necessarily linear.

When applying the proposed trust framework as an extension of an application that already uses two types of data, fewer modifications would be required. For example, QoWater [56] employs a wireless sensor network to monitor water distribution network infrastructure through objective measurements and collects feedback from users about the water quality (subjective measurements about water taste, color, odor, appearance and pressure). In this system, we could apply the proposed trust framework and derive prosumers’ trust attitudes based on comparing their subjective measurements and objective values obtained via wireless sensors. The proposed method for computing the trustworthiness of contributed data could compare and adjust users’ subjective measurements in order to get a more trustworthy overall water quality score. Another example includes Metro Cognition application [57] that is intended to offer citizens personalized travel information to make their journeys more convenient. The application gathers both passive sensory information and active user-generated content. For example, the application collects users feedback about metro delays, such that it asks users how long they have to wait for a metro and the users respond choosing one of the following options: a lot, quite a bit, some time, not at all. In this case, an application of our proposed framework is straightforward. Our framework would improve the interpretation of the collected feedback considering users’ attitudes.

Furthermore, mobile crowdsensing applications that collect one type of data, i.e., sensor measurements via smart devices, are widespread. In these cases, the applications could to be extended with the possibility to collect the subjective values of the observed event in order to apply proposed trust framework. Extensions in terms of creating relevant questionnaires or other ways to submit subjective assessments of events raises questions related to mechanisms for promoting users’ cooperation. In some cases, trust and reputation management mechanisms *per se* are used to incentivize users to contribute trustworthy data, where data trustworthiness and user reputation are used to determine a reward [29,52,58]. Other goals of incentive systems are to make a balance between platform and user utility such that they maximize platform utility (i.e., payments to reputable users contributing useful data and no payments to malicious users who provide bad data), while keeping user utility at a satisfactory level to ensure their participation [59,60,61,62]. Although incentive mechanisms are closely related to trust management in mobile crowdsensing systems, they are not the focus of this work.

Sharing data to an application also raises privacy concerns, taking into consideration that there are many types of attacks on privacy, such as monitoring and eavesdropping, traffic analysis, user identification attacks, sensitive location tracking, sequential tracking attacks, task tracing attacks and location-based inference attacks, for which different solutions have been proposed [63,64,65]. Since privacy and security concerns are not the focus of this work, we assume that participants share data to a trusted application server with secured transmission techniques, as well as that the sensing data is generated by a trusted middleware. We assume that communication between a user’s smartphone and a mobile crowdsensing platform is secure and that privacy and security concerns related to integrity, confidentiality and availability are sufficiently handled [66,67,68]. In our method, we assume that the platform receives accurate and complete data, on the basis of which it can derive precise user behavior profiles/attitudes and compare them with each other. Privacy-preserving techniques that provide incomplete information in return for providing privacy are not used, or used properly [55,69,70,71].

We also consider sensor readings as accurate. Namely, smartphones are presently equipped with powerful hardware and are able to provide accurate sensor data. According to [72], the accuracy of modern smartphone sensor readings is 97–98% for specific sensors, such as accelerometers, while the measurement accuracy of temperature, noise levels or luminosity are affected by the location of the smartphone. The smartphone sensors accuracy and reliability also depend on type of smartphone (or smart device) and which hardware it uses. However, even low cost sensors are able to provide accurate measurements [73]. Additionally, the measurements can be improved if sensors are calibrated frequently. Based on that, in our proposed framework we assume that the sensor measurements with sufficient accuracy are provided and we do not handle sensor measurements errors.

## 5. Conclusions

We have proposed a conceptual trust framework for mobile crowdsensing systems, including a novel method for evaluating the trustworthiness of data contributed by users. The proposed trust formalization takes into account that participants in mobile crowdsensing environments contribute their opinions and other subjective data besides the raw sensing data generated by their smart devices. Our proposed method considers different user behaviors and is based on comparison of their contributed assessments by applying nonparametric statistics. The aim of the proposed method is to compute the trustworthiness of users’ contributions in order to use trustworthy and best fitting assessments only for the computation of the event’s trust. We have evaluated our method with extensive simulations and showed that our method achieves a lower error rate than the referenced approaches, i.e., the method proposed by Huang et al. [23], and the simple average method that does not consider data trustworthiness. In societies with different numbers of malicious users, we improved the error rate by 25.9% on average over Huang’s method and by 31.6% on average over the method where all data are considered equally trustworthy. In societies with different distributions of users’ personality types, our method outperformed Huang’s method by 31.2% on average, and the method where all data are considered equally trustworthy by 35.5% on average. The proposed framework and the method are generic and applicable to real-world use cases in different mobile applications that use opportunistic and participatory sensing approaches.

## Figures and Tables

**Table 1:** Distributions of personality types.

Distribution/Personality Type	Uniform	Bipolar	Moderate	Extreme
⇑ (optimistic)	16.67%	0.00%	0.00%	33.33%
⇓ (pessimistic)	16.67%	0.00%	0.00%	33.33%
∼ (centralistic)	16.67%	0.00%	33.34%	33.34%
↔ (opportunistic)	16.67%	0.00%	0.00%	0.00%
↑ (moderate optimistic)	16.67%	50.00%	33.33%	0.00%
↓ (moderate pessimistic)	16.67%	50.00%	33.33%	0.00%
